# Prophylactic cranial irradiation versus active MRI surveillance for limited-stage small-cell lung cancer achieving response after chemoradiotherapy: a protocol for a randomized, controlled, multicenter, phase III trial

**DOI:** 10.1186/s12931-026-03902-0

**Published:** 2026-09-08

**Authors:** Zhonghui Wei, Yu Chen, Jinming Yu, Xiangjiao Meng

**Affiliations:** 1 Cancer Center, Shandong University, Department of Radiation Oncology, Shandong Cancer Hospital and Institute, Shandong First Medical University and Shandong Academy of Medical Sciences, Jinan, Shandong China; 2 Department of Radiation Oncology, Shandong Cancer Hospital and Institute, Shandong First Medical University, Shandong Academy of Medical Sciences, Jinan, Shandong China

**Keywords:** Limited-stage small-cell lung cancer, Prophylactic cranial irradiation, Magnetic resonance imaging, Overall survival, Neurocognitive function, Randomized controlled trial

## Abstract

**Background:**

Prophylactic cranial irradiation (PCI) is the standard preventive strategy for limited-stage small-cell lung cancer (LS-SCLC). However, the evidence supporting this strategy is largely based on the pre-MRI era. With the advancement of modern MRI and treatment techniques, the survival benefit of PCI remains unclear, particularly given the potential for neurocognitive toxicity to impair quality of life. This study aims to re-evaluate the value of PCI in contemporary LS-SCLC patients and investigate whether active MRI surveillance is non-inferior to PCI regarding overall survival (OS).

**Methods:**

This is a prospective, randomized, multicenter, phase III trial, enrolling patients with LS-SCLC who achieve complete response (CR) or partial response (PR) after platinum-based chemoradiotherapy. Participants will be randomly assigned (1:1) to receive either PCI or active MRI surveillance. The primary endpoint is OS. Secondary endpoints include 1- and 3-year OS rates, progression-free survival (PFS), brain metastasis rate, and neurocognitive function. Exploratory analyses will integrate clinical characteristics with the longitudinal kinetics of tumor biomarkers in collected peripheral blood to identify patient subgroups who are more likely to benefit from PCI.

**Discussion:**

This study will re-evaluate the role of PCI in the MRI era for LS-SCLC. If active MRI surveillance proves non-inferior, it may provide evidence supporting a less intensive management approach that could potentially reduce treatment-related neurotoxicity and preserve quality of life. Furthermore, our trial aims to identify patient subgroups that derive a clinical benefit from PCI, and leverage serial biomarker dynamics to guide personalized clinical management for LS-SCLC patients.

**Trial registration:**

This trial is registered at ClinicalTrials.gov (NCT04829708).

## Introduction

Small-cell lung cancer (SCLC) is an aggressive malignancy with a high propensity for early dissemination, particularly to the brain [1, 2]. The brain metastasis rate at one year can be as high as 28% [3, 4]. For decades, PCI has been recommended for patients with LS-SCLC who respond to first-line chemoradiotherapy, based on a meta-analysis which demonstrated a 5.4% increase in 3-year survival and a significant reduction in brain metastasis incidence [5]. However, the trials included in this meta-analysis were conducted prior to the routine use of MRI for staging and follow-up. The question of whether the PCI examined in this study is truly prophylactic or is actually early treatment for potential asymptomatic brain metastases was extensively discussed.

The evolution of MRI technology has significantly increased the detection rate of intracranial metastases, enabling the identification of subclinical lesions before symptoms occur. A retrospective analysis of LS-SCLC patients indicates that PCI provided no significant benefit over observation regarding 3-year brain metastasis incidence (20.4% vs. 11.2%, *p* = 0.10) or overall survival (*p* = 0.32) [6]. Similarly, another meta-analysis indicated that among patients confirmed free of brain metastases via MRI, PCI conferred no significant overall survival benefit (HR 0.74; 95% CI, 0.52–1.05; *p* = 0.08; *n* = 9 studies; *n* = 1384 patients) [7]. Therefore, the strategy of early intervention for MRI-detected lesions warrants investigation as a potential alternative to PCI in LS-SCLC. Notably, a prospective Japanese trial in ES-SCLC found that PCI offered no survival advantage and was associated with neurocognitive toxicity [8]. Given the lack of randomized controlled data for LS-SCLC, there is a pressing need for prospective research to assess the efficacy and safety of PCI in the era of advanced MRI.

The advent of high-resolution MRI allows for the early detection of limited brain metastases, while advances in stereotactic radiation (SRS/SRT) offer a highly effective and focal treatment option [9–13]. A retrospective study comparing SRS and whole brain radiotherapy (WBRT) in SCLC patients with limited brain metastases revealed no significant difference in prognosis [9]. Furthermore, multiple studies have demonstrated that SRS is associated with significantly lower neurotoxicity compared to PCI and WBRT [10–14]. The integration of MRI and SRS/SRT creates new opportunities for early detection and management of brain metastases in LS-SCLC, potentially preserving both treatment efficacy and quality of life, suggesting that PCI may no longer be an obligatory choice.

Consequently, we launched a randomized, multicenter, phase III study (NCT04829708) to establish prospective evidence comparing the efficacy and safety of PCI against MRI surveillance in LS-SCLC patients with confirmed response to first-line chemoradiotherapy. This study will also assess the long-term neurocognitive effects of PCI and determine if modern hippocampal-sparing techniques offer improved safety and survival outcomes. Additionally, by integrating clinical characteristics with serial analyses of peripheral blood biomarkers, we aim to screen for populations that benefit from PCI, thereby facilitating personalized clinical management.

## Methods

### Study design and setting

This is a prospective, open-label, randomized controlled, multicenter, phase III clinical trial designed to evaluate the efficacy and safety of PCI compared with cranial MRI surveillance in patients with LS-SCLC who have achieved a response (CR or PR) following first-line chemoradiotherapy. The study will be conducted across multiple tertiary hospitals in China with expertise in thoracic oncology, radiation oncology, and neurocognitive assessment.

Eligible participants will be randomized in a 1:1 ratio to either the PCI arm or the MRI surveillance arm. Randomization will be stratified according to response status (CR vs. PR), tumor stage (I/II vs. III), and radiotherapy schedule (concurrent vs. sequential). Study coordination, data collection, and management will be centrally overseen by the sponsor; routine monitoring will ensure data quality and adherence to the protocol. The trial is conducted in accordance with Good Clinical Practice and the Declaration of Helsinki, with written informed consent obtained for all patients. This trial is registered at ClinicalTrials.gov (NCT04829708). Any significant protocol modifications, including changes to eligibility criteria, study endpoints, or statistical analyses, will be submitted to the IRB and updated in the trial registry as required. Protocol version 1.3, finalized in September 2024, was used for this submission.

### Participants

#### Key eligibility criteria

Patients will be eligible for enrollment if they meet all of the following criteria:

(I) Provision of written informed consent;
(II) Age ≥ 18 years and ≤ 75 years at the time of enrollment;
(III) Histologically or cytologically confirmed LS-SCLC (according to the VALG staging system);
(IV) Completion of first-line chemoradiotherapy with radiologically confirmed CR or PR according to RECIST v1.1 [15]; patients who have received immunotherapy as part of first-line treatment are also eligible.
(V) No evidence of brain or meningeal metastases on brain MRI performed within 4 weeks prior to enrollment;
(V1) ECOG performance status of 0 to 2 [16], with no deterioration in the preceding 2 weeks;
(VII) Interval between completion of the last treatment cycle and randomization not exceeding 8 weeks;
(VIII) Life expectancy of ≥ 12 weeks;
(IX) Adequate organ function, defined as absolute neutrophil count ≥ 1.5 × 10⁹/L, platelets ≥ 75 × 10⁹/L, hemoglobin ≥ 9.0 g/dL, normal coagulation function, and adequate hepatic and renal function.

#### Key exclusion criteria are as follows:

(I) Diagnosis of extensive-stage SCLC (ES-SCLC) or combined SCLC;
(II) Confirmed brain or meningeal metastases prior to randomization;
(III) History of other active malignancies within 5 years (excluding curable non-melanoma skin cancer or in situ carcinomas);
(IV) Prior head and neck radiotherapy with field overlap precluding PCI;
(V) Contraindications to MRI or severe, uncontrolled comorbidities (e.g., severe cardiovascular disease, recent stroke within 6 months);
(VI) Pregnant or breastfeeding women.
(VII) Any other condition meeting the general exclusion criteria, including but not limited to severe hematologic, cardiac, hepatic, or renal dysfunction or immunodeficiency.

### Overall study design

Before enrollment, all patients must have completed at least two cycles of chemotherapy and definitive radiotherapy, achieving a confirmed radiological response (CR or PR). The prescribed planning target volume (PTV) radiation dose is 54–66 Gy delivered in 1.8–2.0 Gy once-daily fractions or 45 Gy delivered in 1.5 Gy twice-daily fractions. Baseline imaging, including contrast-enhanced chest and abdominal computed tomography (CT) or positron emission tomography–computed tomography (PET-CT), and brain MRI, will be performed within 4 weeks prior to enrollment to exclude intracranial metastases.Bone scintigraphy is recommended for patients with clinical suspicion of bone metastases.

Eligible patients will undergo randomization within 8 weeks after completion of first-line chemoradiotherapy. Patients assigned to the PCI group will undergo prophylactic whole-brain radiotherapy within 8 weeks after the completion of CRT. The prescribed total dose is 25 Gy delivered in 10 fractions (2.5 Gy per fraction), administered once daily, 5 days per week. Hippocampal avoidance (HA) techniques are recommended to minimize neurocognitive toxicity.

**Fig. 1:**
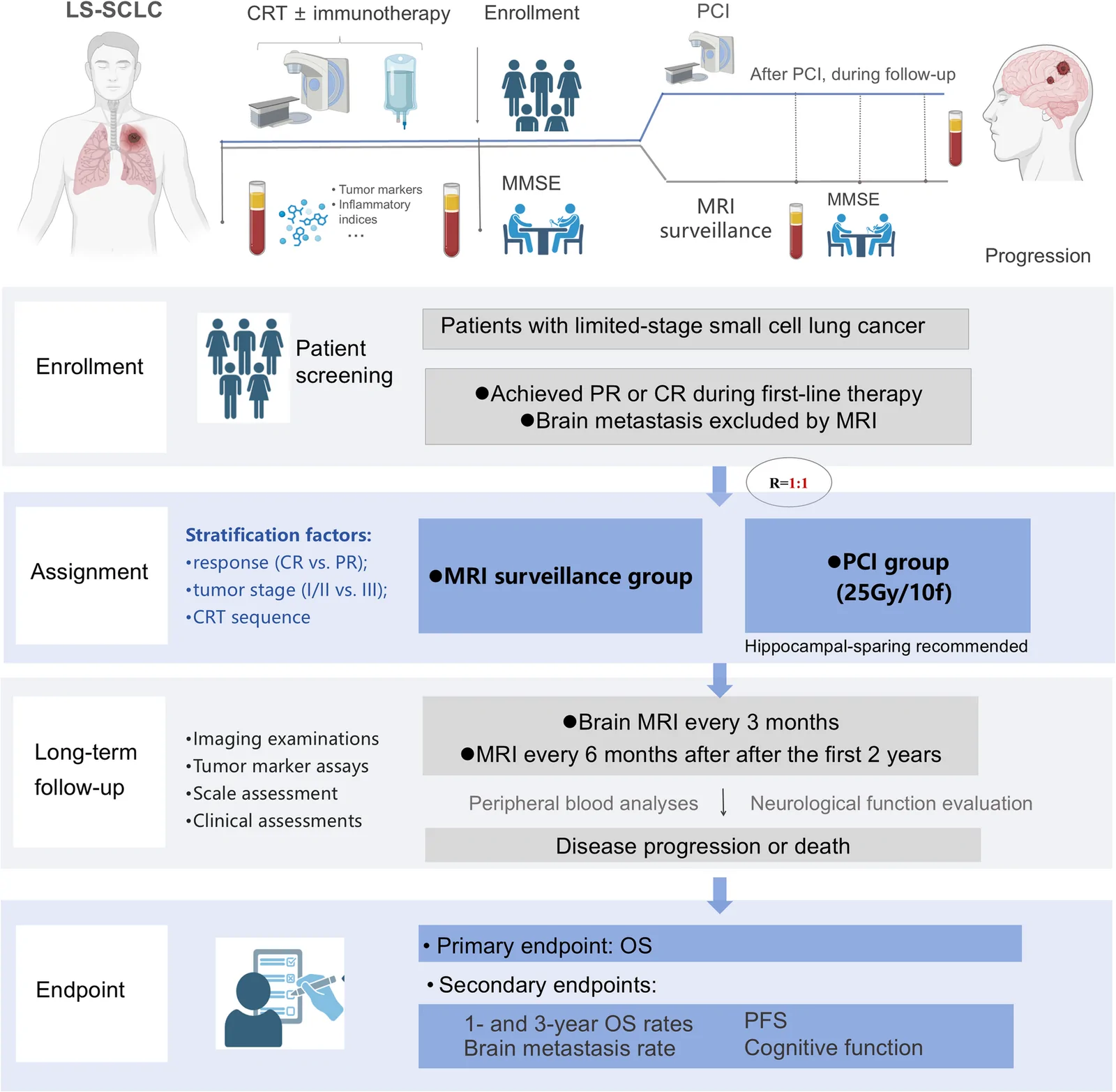
Flowchart of the study. CRT, chemoradiotherapy; CR, complete response; PR, partial response; MMSE, Mini-Mental State Examination

Patients assigned to the MRI surveillance group will undergo active surveillance. Follow-up assessments for both groups will include cranial MRI every no more than 3 months for the first 24 months, and every no more than 6 months thereafter until the detection of brain metastases. Imaging should be performed immediately when symptoms arise, including headache, dizziness, diplopia, nausea, or vomiting. Additionally, contrast-enhanced CT of the neck, chest, and abdomen is recommended, where feasible, to identify any extracranial disease progression or recurrence. For patients who develop brain metastases, subsequent local therapy (such as WBRT or SRS) will be determined by the treating investigator based on the number and size of metastases, patient performance status, and institutional guidelines. Following completion of salvage treatment, patients will undergo surveillance with contrast-enhanced MRI or CT every 2–3 months for the first 24 months, and every 6 months thereafter until the development of subsequent intracranial metastases. A schematic of the study process is shown in Fig. 1.

Both groups will undergo identical schedules for systemic disease evaluation. Follow-up visits will include clinical assessment, toxicity evaluation, imaging, and peripheral blood monitoring as per institutional standards, as well as neurocognitive function assessment. All patients will be followed for survival until death, withdrawal of consent, or study closure.

### Neurocognitive function assessment

To evaluate the potential neurotoxicity associated with PCI compared to surveillance, neurocognitive function will be assessed at pre-specified time points: baseline, and at 3, 6, 12, and 24 months after randomization. Assessments will be performed using the Mini-Mental State Examination (MMSE) scale [17] and a standardized neurological examination checklist [18]. Results will be correlated with quality of life (QoL) outcomes [19].

### Dynamic peripheral blood monitoring

In this trial, peripheral blood is harvested periodically: at baseline, periodically during the follow-up period, and additionally upon disease progression. Tumor marker assessments are performed at every follow-up visit. Follow-up persists until death or withdrawal. By dynamically monitoring inflammatory indices and tumor markers, integrating these longitudinal changes with clinical characteristics, we will employ machine learning models to identify patient subgroups that derive survival benefits from PCI. This approach provides new insights for precision prophylactic strategies against brain metastases in LS-SCLC.

### Endpoints

The primary endpoint is OS, defined as the time from randomization to death from any cause or lost to follow-up. Secondary endpoints include the 1-year and 3-year OS rates, PFS, intracranial metastasis rate, and neurocognitive function changes assessed by MMSE at specified intervals. Progression-free survival (PFS) will be assessed by investigators according to the Response Evaluation Criteria in Solid Tumors (RECIST) version 1.1 criteria, without blinded independent central review. Validated patient-reported outcome measures will be used to assess QoL where feasible. Exploratory analyses will aim to identify specific patient subgroups that derive the benefit from PCI based on clinical characteristics and longitudinal dynamic biomarkers.

### Sample size and statistical power

The sample size calculation is based on the primary endpoint of OS, using a non-inferiority design. Assuming an exponential survival distribution, simulation based on available data indicates that the median OS in the PCI group is approximately 22 months. Considering that MRI-based surveillance may reduce treatment-related toxicities associated with PCI, the median OS in the MRI surveillance group is estimated to be approximately 23.5 months [5, 8, 20]. Based on a non-inferiority margin corresponding to a hazard ratio (HR) of 1.25, with a one-sided alpha of 0.025 and a power of 80%, a total of 387 OS events are required. With an estimated enrollment period of 60 months and an additional follow-up period of 20 months, a total of 544 patients (272 per arm) needs to be enrolled, accounting for a 5% dropout rate. The calculation was performed using PASS 2021 software.

### Data collection and management

All data, including demographic characteristics, disease history, treatment details, imaging results, laboratory tests, adverse events, and patient-reported outcomes, will be prospectively collected via electronic case report forms (eCRFs). Serious adverse events (SAEs) will be reported to the principal investigator and the respective Institutional Review Board (IRB) within 24 h of awareness. To ensure data quality, range checks and logic checks will be built into the eCRF system. Data entry and management will be performed by trained clinical research coordinators under the supervision of the principal investigator. Source data verification will be performed periodically. Participant confidentiality will be maintained, and all stored data will be de-identified.

### Statistical analysis

The OS and PFS will be analyzed using the Kaplan-Meier method, with differences compared using the log-rank test. Hazard ratios (HRs) and corresponding 95% confidence intervals (CIs) will be estimated using Cox proportional hazards models. The cumulative incidence of brain metastasis will be compared using Gray’s test, considering death as a competing risk. Neurocognitive function scores will be analyzed using mixed-effects models for repeated measures to assess changes from baseline over time. Subgroup analyses for OS will be performed using Cox proportional hazards models. All statistical tests will be one-sided for non-inferiority, with a significance level of 0.025. Statistical analyses will be performed using IBM SPSS Statistics (version 29.0) or R software (version 4.2.0).

## Discussion

The role of PCI in LS-SCLC has been established for decades, primarily based on a meta-analysis of trials conducted prior to the advent of high-precision MRI for staging and follow-up [5]. With technological advancements facilitating the early detection and treatment of brain metastases, PCI applicability in the contemporary era is increasingly questioned. This study, a randomized phase III trial comparing PCI with MRI surveillance in LS-SCLC patients who achieve CR/PR after first-line therapy, is designed to address this critical gap in evidence and provide further insights into which populations may derive greater benefit from PCI.

A meta-analysis in 1999 established the role of PCI in SCLC, demonstrating a 5.4% improvement in the 3-year survival rate and a significant reduction in the incidence of brain metastases (58.6% without PCI vs. 33.3% with PCI, *p* < 0.001) among patients after first-line chemoradiotherapy [5]. However, the trials included in this meta-analysis predate the routine use of MRI and were restricted to LS-SCLC patients achieving CR. With the widespread use of modern MRI, recent retrospective studies indicate that PCI continues to reduce the incidence of brain metastases in LS-SCLC patients with CR or PR, but this reduction does not translate into a survival benefit and may compromise quality of life [6, 21, 22]. Growing evidence suggests that PCI is frequently associated with varying degrees of neurocognitive decline, the severity of which is comparable to the neurotoxicity observed following WBRT for overt metastases [5, 8]. Consequently, approximately 40% of eligible patients decline PCI due to concerns regarding neurotoxicity [23, 24]. To provide more definitive evidence, our trial design incorporates longitudinal neurocognitive assessments at multiple time points to delineate the short- and long-term neurocognitive sequelae of PCI and their associations with survival outcomes. Furthermore, given prospective evidence that hippocampal avoidance may preserve the topological organization of brain networks [24, 25], we recommend the use of HA-PCI in this study, investigating whether HA-PCI can mitigate PCI-related neurocognitive toxicity and potentially improve survival outcomes in patients with LS-SCLC.

The value of PCI in the MRI era was first questioned in ES-SCLC. A landmark randomized trial from Japan in ES-SCLC demonstrated that active MRI surveillance without upfront PCI resulted in non-inferior OS compared to PCI [8]. This pivotal finding prompted a revision of international guidelines, which now favor MRI surveillance over PCI for ES-SCLC. The lack of survival benefit in this population is likely attributable to the competing risk of death; patients with ES-SCLC face an excessively high risk of systemic progression and mortality, meaning the intracranial control provided by PCI often lacks sufficient time to translate into an extension of overall survival. Now, the role of PCI in the modern treatment era of LS-SCLC is also being challenged. While PCI reduces brain metastasis rates, its ability to improve survival warrants prospective validation. Prior studies demonstrated that among LS-SCLC patients who did not receive PCI, a substantial proportion presented with a limited number of intracranial lesions at the time of first brain metastasis, thereby offering greater opportunities for treatment with SRS [12, 14]. This feasibility facilitates a novel therapeutic strategy: early identification via active MRI surveillance followed by the timely SRS treatment.

A recent prospective study demonstrated that for patients with SCLC presenting with limited brain metastases and no prior brain irradiation (including PCI), SRS/SRT reduced neurological mortality (11.0% vs. 17.5% at 1 year) compared to historical WBRT controls [14]. Consequently, active MRI surveillance combined with timely SRS/SRT has demonstrated significant value in the management of SCLC, and better preserved neurocognitive function [10–13, 12]. Furthermore, under close MRI-based surveillance, most patients who develop intracranial recurrence after initial SRS present with a limited number of new lesions that are detected early, thereby allowing for continued salvage treatment with SRS/SRT rather than WBRT. To further explore this strategy, our trial will characterize the patterns and burden of brain metastases and subsequent salvage therapies, and evaluate its potential clinical value.

The advent of immunotherapy has further reshaped the treatment landscape of LS-SCLC, with durvalumab consolidation following chemoradiotherapy now established as the new standard of care. The ADRIATIC trial results indicated that while durvalumab was effective whether or not patients undergo PCI, those receiving PCI showed slightly higher 3-year OS rate: 62.1% in the PCI group compared to 50.2% in the non-PCI group (HR 0.75). Grade 3–4 adverse events occurred in 28.4% of the PCI group versus 19.8% in the non-PCI group(19,28). These findings necessitate a reassessment of the utility of PCI within current immunotherapy treatment landscapes. Our randomized study includes patients who have received first-line immunotherapy, particularly those receiving consolidation immunotherapy, aiming to reassess the role of PCI in the modern treatment era and identify patients who may derive benefit from prophylactic irradiation.

Although our trial has been carefully designed, several limitations should be acknowledged. First, this is an open-label study, as blinding of treatment assignment is not feasible given the inherent characteristics of radiotherapy. Second, the choice of local treatment after the detection of brain metastases in the surveillance arm (e.g., WBRT vs. SRS) was decided jointly by patients and investigators, which may introduce heterogeneity in subsequent patient management and clinical outcomes. To minimize its impact, all post-progression treatments will be systematically documented, and prespecified subgroup analyses will be performed. Third, although MMSE was selected because of its simplicity, feasibility, and widespread use in routine clinical practice and previous PCI-related studies, it may have limited sensitivity for detecting subtle cognitive impairment associated with PCI, particularly in executive function, attention, and delayed recall. More sensitive assessment tools such as the Montreal Cognitive Assessment (MoCA), may provide a more detailed evaluation of PCI-related neurotoxicity and should be incorporated into future prospective studies. Finally, the prolonged follow-up required for overall survival assessment may increase the risk of patient attrition. To mitigate this, we will implement centralized data management and active patient engagement strategies to ensure data integrity and follow-up compliance.

Despite these limitations, this trial is designed to address an important clinical question in LS-SCLC. If the MRI surveillance strategy is demonstrated to be non-inferior to PCI, it may represent a more personalized and function-preserving approach for LS-SCLC survivors. In particular, by reassessing the role of PCI in the modern era of immunotherapy, this study may provide clinically relevant insights that align with the evolving treatment landscape of LS-SCLC. The results will provide evidence on whether active MRI surveillance can reduce exposure to the long-term neurotoxic effects of PCI without compromising survival outcomes. Furthermore, exploratory analyses will aim to characterize patient subgroups who may derive greater benefit from PCI, potentially providing insights into more individualized patient selection. In conclusion, this study seeks to clarify the clinical utility of PCI in the modern treatment era and contribute to the optimization of evidence-based strategies for managing brain relapse risk in LS-SCLC [26].

## Data Availability

No datasets were generated or analysed during the current study.

## Abbreviations

LS-SCLC: Limited-stage small cell lung cancer
ES-SCLC: Extensive-stage small cell lung cancer
PCI: Prophylactic cranial irradiation
MRI: Magnetic resonance imaging
OS: Overall survival
PFS: Progression-free survival
CR: Complete response
PR: Partial response
HR: Hazard ratio
CI: Confidence interval
RECIST v1.1: Response Evaluation Criteria in Solid Tumors version 1.1
ECOG: Eastern Cooperative Oncology Group
SRS: Stereotactic radiosurgery
SRT: Stereotactic radiotherapy
WBRT: Whole-brain radiotherapy
SCRT: Sequential chemoradiotherapy
ICRT: Immune-chemoradiotherapy
MMSE: Mini-Mental State Examination
VALG: Veterans Administration Lung Group
PET-CT: Positron emission tomography–computed tomography
IWRS: Interactive Web Response System
HA: Hippocampal avoidance
QoL: Quality of life
eCRF: Electronic case report form
SAE: Serious adverse event
IRB: Institutional Review Board
PASS: Power Analysis and Sample Size
SPSS: Statistical Package for the Social Sciences

## References

[1] van Meerbeeck JP, Fennell DA, De Ruysscher DKM. Small-cell lung cancer. Lancet. 2011;378(9804):1741–55. 21565397 10.1016/S0140-6736(11)60165-7

[2] Siegel RL, Miller KD, Jemal A. Cancer statistics, 2019. CA Cancer J Clin. 2019;69(1):7–34. 30620402 10.3322/caac.21551

[3] Komaki R, Cox JD, Whitson W. Risk of brain metastasis from small cell carcinoma of the lung related to length of survival and prophylactic irradiation. Cancer Treat Rep. 1981;65(9–10):811–4. 6268295

[4] Eagan RT, Frytak S, Lee RE, Creagan ET, Ingle JN, Nichols WC. A case for preplanned thoracic and prophylactic whole brain radiation therapy in limited small-cell lung cancer. Cancer Clin Trials. 1981;4(3):261–6. 6269769

[5] Aupérin A, Arriagada R, Pignon JP, Le Péchoux C, Gregor A, Stephens RJ, et al. Prophylactic cranial irradiation for patients with small-cell lung cancer in complete remission. Prophylactic Cranial Irradiation Overview Collaborative Group. N Engl J Med. 1999;341(7):476–84. 10441603 10.1056/NEJM199908123410703

[6] Chen Y, Wang Y, Ren F, Huang Z, Tan B, Zhao Z, et al. Prophylactic cranial irradiation (PCI) versus active surveillance in patients with limited-stage small cell lung cancer: a retrospective, multicentre study. Respir Res. 2022;23(1):274. 36184624 10.1186/s12931-022-02196-2 PMC9526908

[7] Gaebe K, Erickson AW, Li AY, Youssef AN, Sharma B, Chan KKW, et al. Re-examining prophylactic cranial irradiation in small cell lung cancer: a systematic review and meta-analysis. eClinicalMedicine. 2024;67:102396. 38261885 10.1016/j.eclinm.2023.102396 PMC10796984

[8] Takahashi T, Yamanaka T, Seto T, Harada H, Nokihara H, Saka H, et al. Prophylactic cranial irradiation versus observation in patients with extensive-disease small-cell lung cancer: a multicentre, randomised, open-label, phase 3 trial. Lancet Oncol. 2017;18(5):663–71. 28343976 10.1016/S1470-2045(17)30230-9

[9] Rusthoven CG, Yamamoto M, Bernhardt D, Smith DE, Gao D, Serizawa T et al. Evaluation of First-line Radiosurgery vs Whole-Brain Radiotherapy for Small Cell Lung Cancer Brain Metastases: The FIRE-SCLC Cohort Study. JAMA Oncol. 2020 July 1;6(7):1028–37. 10.1001/jamaoncol.2020.1271 PMC7273318 32496550

[10] Yomo S, Hayashi M. Is stereotactic radiosurgery a rational treatment option for brain metastases from small cell lung cancer? A retrospective analysis of 70 consecutive patients. BMC Cancer. 2015;15:95. 25879433 10.1186/s12885-015-1103-6 PMC4359776

[11] Jo KW, Kong DS, Lim DH, Ahn YC, Nam DH, Lee JI. The role of radiosurgery in patients with brain metastasis from small cell lung carcinoma. J Korean Neurosurg Soc. 2011;50(2):99–102. 22053227 10.3340/jkns.2011.50.2.99 PMC3206286

[12] Ozawa Y, Omae M, Fujii M, Matsui T, Kato M, Sagisaka S, et al. Management of brain metastasis with magnetic resonance imaging and stereotactic irradiation attenuated benefits of prophylactic cranial irradiation in patients with limited-stage small cell lung cancer. BMC Cancer. 2015;15:589. 26275617 10.1186/s12885-015-1593-2 PMC4537586

[13] Li J, Brown PD. The Diminishing Role of Whole-Brain Radiation Therapy in the Treatment of Brain Metastases. JAMA Oncol. 2017;3(8):1023–4. 28056128 10.1001/jamaoncol.2016.5411

[14] Aizer AA, Tanguturi SK, Shi DD, Catalano PJ, Shin KY, Ricca I, et al. Stereotactic Radiosurgery in Patients With Small Cell Lung Cancer and 1–10 Brain Metastases: A Multi-Institutional, Phase II, Prospective Clinical Trial. J Clin Oncol. 2025 Sept;20(27):2986–97. 10.1200/JCO-25-00056 40644657

[15] Eisenhauer EA, Therasse P, Bogaerts J, Schwartz LH, Sargent D, Ford R, et al. New response evaluation criteria in solid tumours: revised RECIST guideline (version 1.1). Eur J Cancer. 2009;45(2):228–47. 19097774 10.1016/j.ejca.2008.10.026

[16] Oken MM, Creech RH, Tormey DC, Horton J, Davis TE, McFadden ET, et al. Toxicity and response criteria of the Eastern Cooperative Oncology Group. Am J Clin Oncol. 1982;5(6):649–55. 7165009

[17] Folstein MF, Folstein SE, McHugh PR. Mini-mental state. A practical method for grading the cognitive state of patients for the clinician. J Psychiatr Res. 1975;12(3):189–98. 1202204 10.1016/0022-3956(75)90026-6

[18] Wen PY, Chang SM, Van den Bent MJ, Vogelbaum MA, Macdonald DR, Lee EQ. Response Assessment in Neuro-Oncology Clinical Trials. J Clin Oncol 2017 July 20;35(21):2439–49. 10.1200/JCO.2017.72.7511 PMC5516482 28640707

[19] Aaronson NK, Ahmedzai S, Bergman B, Bullinger M, Cull A, Duez NJ, et al. The European Organization for Research and Treatment of Cancer QLQ-C30: a quality-of-life instrument for use in international clinical trials in oncology. J Natl Cancer Inst. 1993;85(5):365–76. 8433390 10.1093/jnci/85.5.365

[20] Cheng Y, Spigel DR, Cho BC, Laktionov KK, Fang J, Chen Y, et al. Durvalumab after Chemoradiotherapy in Limited-Stage Small-Cell Lung Cancer. N Engl J Med. 2024;391(14):1313–27. 39268857 10.1056/NEJMoa2404873

[21] Li X, Lu S, Lv J, Guan S, Yan M, Zhu H, et al. The role of prophylactic cranial irradiation in patients with limited-stage small cell lung cancer at different risks of brain metastasis: A multicenter retrospective study. Radiother Oncol. 2025 July;208:110897. 10.1016/j.radonc.2025.110897 40254167

[22] Chen M, Sun Z, Pan J, Xu Y, Wang Y, Chen M, et al. The impact of Prophylactic cranial irradiation on the prognosis of patients with limited-stage small cell lung cancer in the MRI era. Radiat Oncol. 2024;19(1):162. 39543706 10.1186/s13014-024-02557-9 PMC11566379

[23] Rusthoven CG, Kavanagh BD. Prophylactic Cranial Irradiation (PCI) versus Active MRI Surveillance for Small Cell Lung Cancer: The Case for Equipoise. J Thorac Oncol. 2017;12(12):1746–54. 28882584 10.1016/j.jtho.2017.08.016

[24] Giuliani M, Sun A, Bezjak A, Ma C, Le LW, Brade A, et al. Utilization of prophylactic cranial irradiation in patients with limited stage small cell lung carcinoma. Cancer. 2010;116(24):5694–9. 20803612 10.1002/cncr.25341

[25] Candiff O, Belderbos J, Wolf AL, Damen E, van Haaren P, Crijns W, et al. Quality assurance and safety of hippocampal avoidance prophylactic cranial irradiation in the multicenter randomized phase III trial (NCT01780675). J Natl Cancer Cent. 2023 June;3(2):135–40. 10.1016/j.jncc.2023.05.004 PMC11256712 39035727

[26] Colaes R, Blommaert J, Lambrecht M, de Ruiter MB, Pullens P, de Ruysscher D et al. Hippocampal avoidance prophylactic cranial irradiation (HA-PCI) for small-cell lung cancer better preserves white matter networks compared to conventional PCI. Neuro Oncol. 2025 June 21;27(5):1285–96. 10.1093/neuonc/noae271 PMC12187368 39718983

